# Shrimp‐ and mite sensitization in a Swedish study: Influence on allergic disorders and lung function

**DOI:** 10.1002/clt2.12198

**Published:** 2022-10-09

**Authors:** Ida Waern, Magnus Molin, Robert Movérare, Jonas Lidholm, Andrei Malinovschi, Magnus P. Borres, Christer Janson

**Affiliations:** ^1^ Department of Anatomy, Physiology and Biochemistry Swedish University of Agricultural Sciences Uppsala Sweden; ^2^ Thermo Fisher Scientific Uppsala Sweden; ^3^ Department of Medical Sciences: Respiratory, Allergy and Sleep Research Uppsala University Uppsala Sweden; ^4^ Department of Medical Sciences: Clinical Physiology Uppsala University Uppsala Sweden; ^5^ Department of Maternal and Child Health Uppsala University Uppsala Sweden

To the Editor,

1

Shrimp and house dust mite are common causes of allergic disease. Shrimp allergy represents one of the most prevalent food allergies and allergic reactions in response to intake can generate a variety of clinical manifestations, from mild symptoms in the oral mucosa, urticaria to severe anaphylaxis.^1^ The major shrimp allergen is the muscle protein tropomyosin (Pen a 1). Food allergy is associated with severe asthma and increased risk of asthma exacerbations.^2^ In a Swedish study on young asthmatics, 15% were sensitized to shrimp, but only 8% reported shrimp hypersensitivity.^3^

The indoor allergen house dust mite is a major cause of allergic conditions, including rhinitis and asthma, and one of the most common allergens to which asthmatic patients are sensitized.^4^ In addition to several house dust‐mite specific allergens, tropomyosin has been identified as an allergen in house dust mites.^5^ The muscle protein tropomyosin is not only expressed in shrimp and house dust mite, but homologous forms are also found in insects and molluscs. Therefore, it is likely that patients sensitized to shrimp and house dust mites might be at risk for cross‐reactions to allergens from other species. In addition, other panallergens than tropomyosin have been associated with cross allergies.^1^ Allergic reactions mediated by cross‐reactive IgE can occur in individuals primarily sensitized to airborne allergens, who also show sensitivity to proteins present in food. Broekman et al. have shown that IgE from shrimp allergic patients recognized proteins from insect extracts.^6^

In this study, we aimed to assess the prevalence of shrimp and house dust mite sensitization in a large population‐based study and to compare IgE sensitization in relation to allergic disorders and respiratory symptoms. Chi‐squared test and ANOVA was used to detect group differences in the univariate analyses, while logistic and linear regression was used in adjusted analyses.

A total of 4593 randomly selected adult subjects aged 50–64 years from the Swedish CArdioPulmonary bioImage Study (SCAPIS), Uppsala cohort, took part in an extensive questionnaire, blood sampling, physical examinations, lung function tests and imaging.^7^, ^8^ IgE sensitization was defined as having specific IgE ≥0.35 kU_A_/L, measured by ImmunoCAP (Thermo Fisher Scientific). Forced expiratory volume in 1 s (FEV_1_) and forced vital capacity after bronchodilation was measured. Chronic airflow limitation (CAL) was defined as having an FEV_1_/FVC ratio below 0.70.

Of all participants, 253 (5.5%) were IgE sensitized to shrimp (≥0.35 kU_A_/L), 191 (4.2%) were mite‐sensitized, and 104 (2.3%) were sensitized to both allergens. Of the shrimp sensitized patients, 41% were sensitized to mite, whereas 54% of the mite sensitized patients were sensitized to shrimp. There was no significant association between the specific IgE titer against mite and being sensitized to shrimp in those that were mite sensitized (*p* = 0.37).

Participants were divided into four groups based on their sensitization to shrimp, house dust mite, shrimp and house dust mite or neither of them. The characteristics of these groups are presented in Table 1. Significant group differences were found regarding the prevalence of asthma, allergic rhinitis, urticaria, angioedema, wheeze, and CAL.

**TABLE 1:** Characteristics of study groups defined by sensitization to shrimp and/or house dust mite (% and mean ± SD) and logistic regression adjusted for age, sex, and sensitization to birch and cat (odds ratio [95% confidence interval])

	Non‐sensitized (shrimp or mite) (*n* = 4253)	Only shrimp‐sensitized (*n* = 149)	Only mite‐sensitized (*n* = 87)	Sensitized to both shrimp and mite (*n* = 104)	*p* value
Women (%)	52.6	38.3	43.7	43.3	0.001
Age (yrs)	57.7 ± 4.4	57.9 ± 4.2	56.9 ± 4.8	57.3 ± 4.6	0.27
Asthma (%)	5.7	5.0	17.1	16.5	<0.0001
Allergic rhinitis (%)	19.9	28.3	46.2	34.8	<0.0001
Urticaria (%)	26.5	35.6	44.9	36.4	<0.0001
Angioedema (%)	5.2	9.7	11.4	13.2	<0.0001
Wheeze (%)	6.4	5.0	9.9	16.3	0.001
FEV_1_% predicted (%)	109 ± 14	109 ± 15	110 ± 13	105 ± 15	0.02
Chronic airflow limitation (%)	7.4	4.8	6.1	15.2	0.02

	OR (95% CI)	OR (95% CI)	OR (95% CI)	OR (95% CI)	
Asthma	1	0.60 (0.27–1.34)	1.82 (0.95–3.48)	1.67 (0.88–3.17)	
Allergic rhinitis	1	1.01 (0.65–1.56)	1.64 (0.97–2.80)	0.91 (0.54–1.53)	
Urticaria	**1**	**1.55 (1.05‐2.28)**	**2.19 (1.33‐3.60)**	1.49 (0.93–2.41)	
Angioedema	1	1.66 (0.88–3.86)	1.83 (0.87–3.86)	**2.28 (1.18‐4.39)**	
Wheeze	1	0.70 (0.32–1.52)	1.36 (0.63–2.92)	**2.47 (1.36‐4.47)**	
Chronic airflow limitation	1	0.57 (0.26–1.24)	0.81 (0.32–2.05)	**2.03 (1.11‐3.71)**	

Being sensitized to both shrimp and mite was independently associated with having angioedema, wheeze, and chronic airflow limitation after adjusting for age, sex, birch, and cat sensitization. Being sensitized to mite alone was associated with having urticaria (Table 1). There was also an independent association between lower FEV_1_ (% of predicted) and being sensitization to both shrimp and mite: −3.5 (−6.5, −0.5)% of predicted units.

The main finding of this study is that approximately half of those with IgE sensitization to shrimp were also sensitized to mite and vice versa. We also found that participants that were sensitized to both shrimp and mite were more likely to have respiratory symptoms, angioedema and airflow limitation.

Our results suggest that patients presenting symptoms of shrimp or mite allergy should be examined for sensitization and allergy to both allergens. Our results are also relevant when new food products such as insects are introduced in our society because insects express similar proteins, for example, the muscle protein tropomyosin, which is similar to tropomyosin allergens found in shrimp and mites.

## FUNDING INFORMATION

Swedish Research Council Formas; 2017‐00818. 10.13039/501100003793; Hjärt‐Lungfonden

## CONFLICT OF INTEREST

Magnus Molin, Robert Movérare, Jonas Lidholm and Magnus P. Borres are employed by Thermo Fisher Scientific. Ida Waern, Andrei Malinovschi and Christer Janson have no conflicts of interest to declare.

## Supporting information

Supporting Information S1
