# Supplementary material for: Shrimp‐ and mite sensitization in a Swedish study: Influence on allergic disorders and lung function
Source: Clin Transl Allergy. 2022 Oct 9;12(10):e12198. doi: 10.1002/clt2.12198 (PMC9549068; doi:10.1002/clt2.12198)
Supplement: Supplementary file 1 — Supporting Information S1 [file CLT2-12-e12198-s001.docx]

**Supporting information**

**Materials and methods**

**Study population**

The multicentric Swedish CArdioPulmonary bioImage Study (SCAPIS) consisted of 4958 randomly selected men and women aged 50-64 years. Participants filled in a comprehensive questionnaire and underwent extensive physical examination, lung function tests and blood sampling. Specific IgE measurement was an add-on parameter in Uppsala, one of the six participating SCAPIS sites and full IgE data was available for 4593 subjects^1^.

**Ethics**

The study was approved by the Regional Ethics Review Board in Umeå (2010-228-31M). The allergic analyses sensitization analyses and the present study were approved by the Regional Ethical Review Board in Uppsala (2018-272).

**Questionnaire**

Asthma: Have you ever had asthma diagnosed by a doctor? If yes- do you still have asthma?

Allergic rhinitis: Have you been troubled by nasal allergy during the last 12 months?

Urticaria; Have you ever had urticaria (Pruritic, light red, elevated rash on the body)”?

Angioedema: Have you ever had Quincke’s edema/angioedema (Sudden swelling in your face, throat, or extremities)?

Wheeze: Have you had wheezing or whistling in your chest at any time in the last 12 months?

**Specific IgE testing**

Serum IgE antibodies to shrimp was measured using ImmunoCAP™ Specific IgE (Phadia AB/Thermo Fisher Scientific) and IgE antibodies to inhalant allergens were identified using the ImmunoCAP Phadiatop™ test. The Phadiatop test includes a mix of common perennial and seasonal aeroallergens. Phadiatop-positive sera were re-analyzed for IgE antibodies to house dust mite (*Dermatophagoides pteronyssinus*), cat dander and birch pollen using ImmunoCAP (reflex testing). Sensitization to the specific allergen was defined as an IgE value of ≥0.35 kU_A_/L.

**Lung function**

Dynamic spirometry (Jaeger MasterScreen™ PFT; Carefusion, Hoechberg, Germany) was performed 15 min after bronchodilation of 400 μg salbutamol administered via spacer, with subjects in the sitting position and wearing a nose clip. FEV_1_ and FVC were obtained according to the ATS and ERS standards^2^. Reference equations by Quanjer et al. were used to calculate FEV_1_ as % of the predicted value^3^.

**References**

1. Zaigham S, Zhou X, Molin M, Sjölander A, Movérare R, Janson C, Malinovschi A. Importance of type and degree of IgE sensitisation for defining fractional exhaled nitric oxide reference values. *Respir Med.* 2021;188:106621.
2. Miller MR, Hankinson J, Brusasco V, Burgos F, Casaburi R, Coates A, Crapo R, Enright P, van der Grinten CPM, Gustafsson P, Jensen R, Johnson DC, MacIntyre N, R. McKay, Navajas D, Pedersen OF, Pellegrino R, Viegi G, Wanger J.

Standardisation of spirometry. *Eur Resp J.* 2005 26: 319-338.

1. Quanjer PH, Tammeling GJ, Cotes JE, Pedersen OF, Peslin R, Yernault JC. Lung volumes and forced ventilatory flows. Report Working Party Standardization of Lung Function Tests, European Community for Steel and Coal. Official Statement of the European Respiratory Society. *Eur Respir J Suppl*. 1993;16:5-40
